# Divergent impacts of the neonicotinoid insecticide, clothianidin, on flight performance metrics in two species of migratory butterflies

**DOI:** 10.1093/conphys/coae002

**Published:** 2024-02-02

**Authors:** Staci Cibotti, Phineas J Saum, Andrew J Myrick, Rudolf J Schilder, Jared G Ali

**Affiliations:** Department of Entomology, The Pennsylvania State University, 501 Agricultural Science and Industries Building, University Park, PA 16802, USA; Department of Entomology, The Pennsylvania State University, 501 Agricultural Science and Industries Building, University Park, PA 16802, USA; Department of Entomology, The Pennsylvania State University, 501 Agricultural Science and Industries Building, University Park, PA 16802, USA; Department of Entomology, The Pennsylvania State University, 501 Agricultural Science and Industries Building, University Park, PA 16802, USA; Department of Biology, Pennsylvania State University, University Park, PA 16802, USA; Department of Entomology, The Pennsylvania State University, 501 Agricultural Science and Industries Building, University Park, PA 16802, USA

**Keywords:** Energetics, flight, Lepidoptera

## Abstract

Long-distance flight is crucial for the survival of migratory insects, and disruptions to their flight capacity can have significant consequences for conservation. In this study, we examined how a widely used insecticide, clothianidin (class: neonicotinoid), impacted the flight performance of two species of migratory butterflies, monarchs (*Danaus plexippus*) and painted ladies (*Vanessa cardui*). To do this, we quantified the free-flight energetics and tethered-flight velocity and distance of the two species using flow-through respirometry and flight mill assays. Our findings show differential effects of the pesticide on the two species. For painted ladies, we found that clothianidin exposure reduced average free-flight metabolic rates, but did not affect either average velocity or total distance during tethered flight. Other studies have linked low flight metabolic rates with reduced dispersal capacity, indicating that clothianidin exposure may hinder painted lady flight performance in the wild. Conversely, for monarchs, we saw no significant effect of clothianidin exposure on average free-flight metabolic rates but did observe increases in the average velocity, and for large individuals, total distance achieved by clothianidin-exposed monarchs in tethered flight. This suggests a potential stimulatory response of monarchs to low-dose exposures to clothianidin. These findings indicate that clothianidin exposure has the potential to influence the flight performance of butterflies, but that not all species are impacted in the same way. This highlights the need to be thoughtful when selecting performance assays, as different assays can evaluate fundamentally distinct aspects of physiology, and as such may yield divergent results.

## Teaser

We examined how a widely applied insecticide, clothianidin, impacted the flight performance of two migratory butterfly species, monarchs and painted ladies, using respirometry and flight mill assays. Clothianidin affected both species differentially, with painted ladies showing evidence of some impaired flight traits, while monarchs displayed more stimulatory responses to exposure.

## Introduction

Dispersal is a fundamental biological process with widespread implications for individual fitness, population dynamics, population genetics and species distributions (Kokko and López-Sepulcre, 2006). As such, anthropogenic stressors that impact an organism’s dispersal capacity are likely to carry major consequences for the stability of populations and ecosystems. Farmland occupies >895 million acres in the USA (USDA, 2022), which has resulted in the fragmentation of natural ecosystems and, in many cases, the leaching of toxic agrochemicals, such as pesticides, into the environment. Pesticides can impact the dispersal capacity of a wide range of non-target taxa, including various birds and fishes (Geluso *et al*., 1976; Aktar *et al*., 2009; Macneale *et al*., 2010; Moye and Pritsos, 2010; Eng *et al*., 2017). However, the omnipresence of broad-spectrum insecticide usage on farmland throughout the USA elicits particular concern over their potential effects on non-target insect species (Wood and Goulson, 2017; DiBartolomeis *et al*., 2019; Douglas *et al*., 2020; Serrão *et al*., 2022).

Neonicotinoids are the most widely used class of insecticides in the world (Simon-Delso *et al*., 2015). Their water solubility, long half-lives, and potent broad-spectrum toxicity are all characteristics that make them effective pesticides; however, these same features have raised concern regarding their non-target impacts on beneficial insect species. Recent studies have revealed negative correlations between neonicotinoid usage and butterfly abundances and population indices in several species (Gilburn *et al*., 2015; Pelton *et al*., 2019), indicating that widespread use of these compounds may be harmful to butterfly populations. Neonicotinoids target nicotinic acetylcholine receptors in the insect nervous system, which are involved in the regulation of learning and motor function (Colquhoun *et al*., 1987). Insects that have been acutely exposed to these compounds often exhibit muscle spasming (Camp and Buchwalter, 2016), and this short-term muscle hyperactivity could lead to long-term muscle exhaustion and/or energetic depletion, both of which could have negative consequences for insect flight capacity. Studies evaluating the effects of neonicotinoids on honeybee flight have found that exposure to sublethal doses of neonicotinoids can reduce their flight ability, foraging capacity and ability to successfully complete homing flights (Matsumoto, 2013; Fischer *et al*., 2014; Tosi *et al*., 2017). Given that these compounds can negatively impact insect flight performance, even sublethal exposures could carry major consequences for the dispersal potential and/or migratory capacity of insect species.

Arguably one of the most iconic migratory insects in North America is the monarch butterfly (*Danaus plexippus*, family: Nymphalidae), which can travel up to 5000 km each fall, traversing vast acreage of farmland across southern Canada and the USA to reach their overwintering sites in central Mexico and coastal California (Brower, 1995). Unfortunately, over the past several decades, the number of monarchs successfully completing these migratory journeys has dwindled. Population counts at overwintering sites have revealed a >80% decline in the eastern population, and a >99% decline in the western population since censuses began in the 1990s (Pelton *et al*., 2019). While some believe the major driver of these declines is a reduction in the availability of their obligate host plant, milkweed (*Asclepias spp.*), due to the rise of Round-up® usage (Hartzler, 2010; Pleasants and Oberhauser, 2013; Flockhart *et al*., 2015), others argue that discrepancies between the seemingly stable summer breeding populations and the sharply declining overwintering counts could point to problems encountered during the species’ non-reproductive migratory and/or overwintering stages (Inamine *et al*., 2016; Agrawal and Inamine, 2018).

Another nymphalid butterfly with impressive migratory potential is the painted lady (*Vanessa cardui,* family: Nymphalidae), which can migrate up to three times as far as the monarch butterfly (up to 15 000 km) (Chowdhury *et al*., 2021). In contrast to monarch butterflies, which are milkweed specialists, painted ladies are broad generalists, a characteristic which has contributed to them being the most widely distributed butterfly species in the world (Shields, 1992). Additionally, while the migratory populations of the monarch butterfly were recently listed as endangered by The International Union for the Conservation of Nature (IUCN), all painted lady populations are generally regarded as stable, being classified as a species of least concern by the IUCN (IUCN, 2022).

Given the ecological importance of flight for both species, we sought to investigate whether exposure to neonicotinoid insecticides would affect the flight performance of the two butterflies. To do this, we fed adult butterflies sugar solutions spiked with the widely applied neonicotinoid active ingredient, clothianidin, and then evaluated their flight performance in two separate flight assays. First, we used flow-through respirometry to quantify free-flight energetics (measured as CO_2_ exchange rates) and then we used flight mills to quantify tethered-flight velocity and distance. Given that neonicotinoid insecticides are known to impair flight performance metrics in bees (Matsumoto, 2013; Fischer *et al*., 2014; Tosi *et al*., 2017), we hypothesized that clothianidin exposure would lower the butterflies’ average flight metabolic rates and reduce their total flight distances and average flight velocities. Furthermore, because neonicotinoids are broad-spectrum insecticides, we predicted that both butterfly species would exhibit similar reductions in flight performance in response to clothianidin exposure.

## Materials and Methods

### Insect preparation

We captured 50 wild *D. plexippus* butterflies across several sites near State College, PA, USA between July and August 2019 and used these individuals to establish a laboratory colony that was maintained year-round at the Pennsylvania State University (University Park, PA, USA) on a diet of *Asclepias syriaca* and *Asclepias curassavica*. Upon capture, each monarch was checked for spores of the protozoan parasite, *Ophryocystis elektroscirrha* (OE), and only individuals with no spores present on their bodies were integrated into the colony. Each successive generation was also monitored to ensure the colony remained parasite-free. Colony adults were kept in large mesh cages (160 cm length × 60 cm width × 60 cm height) in a 29°C greenhouse under metal halide lights set to a 16–8 hour light–dark cycle and allowed to mate freely. To collect eggs, we placed potted milkweed plants (either *A. syriaca* or *A. curassavica*) inside of the cage for females to oviposit onto. After 2 days, we removed the plants from the cage, clipped the milkweed tissue where eggs were present, and placed the egg-covered tissue into clear plastic containers of various sizes. These containers were moved to the laboratory and placed under florescent grow lights set to a 16–8 hour light–dark cycle. The temperature underneath the lights averaged ~24–26°C. Once the eggs hatched, the caterpillars were transferred to individual clear plastic 266-ml lidded cups. We cleaned the cups and replenished the leaf material daily until pupation. Once the monarchs pupated, we cleaned the cups again and continued to monitor them daily. After eclosion, we checked the butterflies for OE before returning them to the mesh cages in the greenhouse.

The monarch individuals used in the respirometry portion of this study were from the second generation of our laboratory colony, and those used in the flight mill study were from the fifth generation. In both cases, a subset of the collected eggs was moved to a different area of the laboratory with the same light and temperature conditions. These individuals were reared using the same methods as the rest of the colony up until eclosion. Once eclosed, these butterflies were checked for OE before being randomly assigned to their treatment group. The sex of each butterfly was determined by checking for the presence of the males’ characteristic black spots on the hind wings. They were then assigned a unique identification number that was marked on their right forewing using a fine point permanent marker. We kept males and females in separate mesh cages (39.25 × 39.25 × 59 cm) to ensure that the butterflies remained unmated for the duration of the experiment. The cages were held under the same light and temperature conditions that the butterflies had been reared under. Because the butterflies had not been exposed to fall-like conditions during rearing, the monarchs used in this experiment were not in a state of reproductive diapause (i.e. not the migrant phenotype). Each individual was only removed for data collection purposes (i.e. when receiving a feeding treatment or undergoing a flight assay). They were promptly returned to their assigned cages once the required data was collected. At the end of the experiment each butterfly was flash-frozen in liquid nitrogen and stored at −80°C.

Early instar *V. cardui* larvae were purchased from Ward’s Science® (Ward’s Natural Science, Rochester, NY, USA) in late May 2019 to be used in the respirometry portion of the study and Carolina® (Carolina Biological Supply Company, Burlington, NC, USA) in late May 2020 for use in the flight mill experiment. In both cases, the larvae were fed Ward’s Science® Painted Lady Butterfly Media (Ward’s Natural Science, Rochester, NY, USA), which was prepared in accordance with package instructions. The adults that emerged from the purchased larvae were then used in the respective flight assays.

While the diet’s influence on the outcomes of this experiment for each species is worth considering, we note that Monarchs were provided with milkweed tissue, whereas Painted Ladies were supplied with media. These dietary variations could conceivably influence responses to pesticides. Furthermore, we did not investigate the potential effects of diverse diets—whether media-based or linked to different plant species—on the adults’ interactions with pesticides. Subsequent research should address such intricate interactions. However, it fell beyond the purview of the present study.

All caterpillars were reared in the laboratory under florescent grow lights set to a 16–8 hour light–dark cycle with an average temperature of ~24–26°C. Each individual was kept in a separate 30-ml clear plastic lidded cup and checked daily. Once they pupated, the pupae were transferred to 266-ml clear plastic lidded cups to ensure they had enough space for eclosion. After the adult butterflies emerged from their pupal casing, they were assigned a unique identification number that was labeled on their right forewing using a fine point permanent marker. Because it is difficult to determine the sex of painted lady butterflies without the aid of a dissecting microscope or other magnification, each individual butterfly was kept inside of their own 473-ml clear plastic container to prevent mating. Like with the monarchs, the painted ladies were only removed for data collection purposes and were promptly returned to their assigned containers once the required data was collected. After the experiment concluded, the butterflies were flash-frozen in liquid nitrogen and stored at −80°C. Their sex was later determined by looking for the presence of setae on the forelegs (indicative of a female) under a dissecting microscope.

### Respirometry experiment

To examine how clothianidin exposure affected the average flight metabolic rates of monarch and painted lady butterflies, we first subjected the butterflies to controlled feeding treatments. To begin, 60 monarch and 46 painted lady butterflies were randomly assigned to one of three pesticide treatments: control (no clothianidin), low dose (0.5 ng/g clothianidin) or high dose (1.5 ng/g clothianidin) (monarch: 20 control, 20 low dose, 20 high dose | painted lady: 16 control, 15 low dose, 15 high dose). The doses were chosen to reflect a range of clothianidin concentrations found in the nectar of wildflowers grown in agricultural field margins (Mogren and Lundgren, 2016).

All butterflies were allowed to feed *ad libitum* on unaltered 20% sugar solutions for the first 5 days post-eclosion. On day 6 post-eclosion, the feeding dishes were removed from the cages and the butterflies were starved for 24 hours to ensure willingness to consume hand-fed treatment solutions. Each day over a 4-day period (days 7–10 post-eclosion), the butterflies were weighed and hand-fed as follows. Immediately preceding their hand-feeding, individual butterflies were weighed inside of glassine envelopes using a Mettler AJ100 balance (Mettler Toldeo, Greifensee, Switzerland), with a 0.1-mg readability. Once their mass was recorded, we removed the butterfly from the envelope, held their wings together, and gently unfurled their proboscis into a pre-assigned tube of treatment solution using a mounting pin. We then held their proboscis submerged in the treatment solution for 3 seconds before removing the pin, leaving the proboscis in place in the tube. The butterflies were allowed to feed uninterrupted until they recoiled their proboscis from the tube. Once recoiled, we waited 5 seconds before gently reintroducing the proboscis back to the treatment solution. We then repeated this process once more, ultimately giving each individual three chances to feed. After they withdrew their proboscis the third time, the butterflies were returned to their glassine envelopes and re-weighed. The difference between their pre- and post-feeding weight was then used to calculate the total clothianidin consumption.

Next, we used flow-through respirometry to assess the average metabolic rates of each individual over 5 minutes of flight on day 7 (after the first clothianidin exposure), and again on day 10 (after 4 consecutive days of clothianidin exposure). This age range was chosen to minimize age-based variation in metabolic rates both among the pool of individuals and between evaluations of the same individual (Woods Jr *et al*., 2010). The average metabolic rate of each individual was quantified 1–2 hours after feeding, and all assessments occurred between the hours of 8:30 and 18:00. In total, four butterflies (3 monarchs and 1 painted lady, all from the control groups) expired over the course of the experiment. In each case, mortality occurred after day 7, but prior to day 10, so only one respirometry trial was captured for these individuals.

We followed the same flow-through respirometry design as Pocius *et al*. (2022). To generate CO_2_-free air for the respirometer, we used a Whatman Ft-IR purge gas generator (Whatman International Ltd, Maidstone, UK) and further scrubbed the air of any residual CO_2_ and H_2_O by passing it through a Drierite/Ascarite/Drierite scrubber column. A 24 V DC circulation pump then pushed the scrubbed air through two chambers, a flight chamber, constructed from a 1.9-l clear polyethylene terephthalate (PET) plastic jar that contained an individual butterfly, and an empty reference chamber of the same design. We maintained a 1 l min^−1^ flow rate into each chamber using a Sable Systems FB-8 (Sable Systems, North Las Vegas, NV, USA) and dual mass flow control valves (Brooks 5850E Mass Flow Controller, Coastal Instruments, Burgaw, NC, USA). Both air lines first emptied into separate manifolds before being subsampled by an SS4 subsampler (Sable Systems, North Las Vegas, NV, USA) and pulled through a LiCOR 7000 CO_2_/H_2_O analyser and an Oxzilla II differential O_2_ analyser (Sable Systems, North Las Vegas, NV, USA) at a rate of 400 ml min^−1^. Both the flow rate and CO_2_ exchange rates were continually monitored and recorded throughout the experiment with a UI2 data acquisition system and ExpeData software (Sable Systems, North Las Vegas, NV, USA).

The flight and reference chambers were contained within a Percival incubator (Percival I-41 L; Percival, Perry, IA, USA), which was set to 28°C and lit with a UV blacklight to encourage flight initiation and sustained flight inside of the chamber (Niitepõld *et al*., 2009; Pocius *et al*., 2022). Before the metabolic rate of each butterfly was assessed, we recorded the CO_2_ levels in our CO_2_-scrubbed air for several minutes to evaluate the baseline CO_2_ levels in the chambers. Then, a single butterfly was placed inside of the flight chamber, and a black cloth was wrapped around the chamber to create a dark environment, which minimized butterfly movement. Once the CO_2_ level inside the chamber stabilized (typically ~10 minutes after the butterfly was introduced), we recorded for an additional minute so that the butterfly’s resting metabolic rate could be calculated (i.e. by subtracting the CO_2_ baseline prior to butterfly introduction from the CO_2_ baseline post-introduction). The resting metabolic rate was then used to establish a baseline comparison for flight metabolic rate. To assess flight metabolic rate, we removed the black cloth and continuously disturbed the chamber to motivate flight over a 5-minute period of evaluation. After 5 minutes, the chamber was re-wrapped in the black cloth so that the butterfly would return to a state of rest. Once at rest, we recorded the post-flight CO_2_ level inside of the chamber for an additional minute before removing the butterfly.

The flight metabolic rate of each butterfly was averaged across the 5-minute flight period, so any changes in activity during the flight period could be accounted for. The raw CO_2_ measurements (recorded in ppm) were Z-transformed to approximate instantaneous gas exchange signals (Bartholomew *et al*., 1981; Pendar and Socha, 2015), and IGOR Pro software (Wavemetrics, Inc., Lake Oswego, OR, USA) was used to convert them into emission rates (ml CO_2_ hr^−1^) for analysis.

### Flight mill experiment

We used flight mills to quantify the total flight distances and average velocities of monarch and painted lady butterflies chronically exposed to clothianidin-contaminated sugar solutions. Twenty-one monarchs and 26 painted lady butterflies were randomly assigned to either a control or a 10 ng/g clothianidin treatment (monarch: 10 control, 11 clothianidin | painted lady: 14 control, 12 clothianidin). The concentration utilized in the respirometry experiment was higher due to a single dose being administered and the extended duration of the experiment. However, it remained within the range of concentrations encountered by butterflies in their natural field environments. (Blacquière *et al*., 2012). All butterflies were fed and weighed following the same protocol as above; however, in this experiment each butterfly was only flown once on day 10 post-eclosion (no flight assessment occurred on day 7), as the flight mill assay was too strenuous to subject the individuals to it twice.

To tether the butterflies to the flight mills we glued a small metal tab (1 mm width × 4 mm length × 5 mm height | ~30 mg weight) to the top of the thorax, which connected to a square magnet (3 mm^3^) on the arm of the mill. To attach the metal tab to the thorax, we first placed the butterflies in glassine envelopes and chilled them in a 4°C cold room for 2 minutes to reduce their mobility to prevent wing injury during the gluing process. Once the butterflies were chilled, we splayed their wings on a foam spreading board, laid pieces of glassine paper over the wings, and pinned the paper down around the wing margin, careful not to puncture or damage the wings. With the wings secured away from the thorax, we were able to remove a small patch of scales from top of the thorax with sandpaper and adhere the metal tab with Loctite® gel super glue (Henkel North American Consumer Goods, Stamford, CT, USA). This metal tab connected to a magnet on the arm of the flight mill, keeping the butterfly secured to the mill for the duration of the flight experiment (see Supplementary Figure 1). Each flight mill was surrounded by an opaque, 56-cm tall tube-shaped plastic sheet, which was held in place with two wooden embroidery hoops, each measuring 64 cm in diameter. The plastic sheets had 8-cm black and white vertical striping that helped to reduce draft, minimize external stimuli and provide the butterfly with a sense of optical flow during flight (see Supplementary Figure 1C). Individual butterflies were affixed to the flight mills 1 hour after feeding, and their flight velocity and distance were recorded uninterrupted for a 12-hour period. This amount of time was chosen because we were interested in flying the butterflies to the point of exhaustion. It has been estimated that the average monarch butterfly could engage in sustained flapping flight for ~11 hours before depleting their energy reserves and needing to stop to refuel. We then rounded up by an hour to account for butterflies that may have higher than average lipid stores. Although these estimates are likely different for painted lady butterflies, we opted to fly both species for the same amount of time for consistency. All of the flight mill assays occurred between the hours of 8:30 and 21:45 and were conducted under fluorescent lights. Although the lighting in the room was not optimized to the species’ critical flicker frequency, all butterflies were flown under the same light conditions, meaning that any potential impacts of flicker frequency on their tethered-flight performance should be equally reflected across the treatment groups.

The flight mills used were of a custom design (see Supplementary Figure 1), having an adjustable turn radius with a maximum of 9.5 cm. The flight arm was attached to a pair of 12.7-mm-outer diameter ball bearings that rotated about a stationary 6.35-mm-diameter stainless steel axis that was mounted to an aluminum base. The arm of the mill was constructed using 2-mm-diameter carbon fiber rod material to minimize its rotational inertia. During flights, a small plate attached to the rotating arm assembly and opposite the flight arm interrupted an infrared LED/detector pair so that turns could be counted. The output of the detector was routed to a Mega 2560 board (Arduino, Somerville, MA, USA) running custom firmware that sent interrupt times to a PC running a custom program written for Labview (National Instruments, Austin, TX, USA) with a resolution of 1 ms and was written to disk. The Mega 2560 and Labview programs were capable of recording from up to 15 flight mills in parallel.

***Data Analysis:*** We analysed the mass-corrected average flight metabolic rates of each species using generalized linear mixed effect models in JMP Pro version 16 (SAS Institute Inc., Cary, NC, USA). Upon reviewing the data on the calculated exposure levels of each individual, we noticed that due to differences in feeding rates, after 4 days of clothianidin feeding, some individuals had total exposure levels that were equal to or below what other individuals of the same species had after just a single clothianidin feeding (see Supplementary Figure S2). We also observed a similar situation between our high and low treatment doses, where some individuals assigned to the low-dose treatment had higher amounts of exposure than others in the high-dose treatment (see Supplementary Figure S2). Because of this, we decided to remove these imposed categorizations and instead analyse all of the data for each species together, using the calculated total amount of clothianidin consumed by each individual prior to their metabolic rate assessment as a fixed effect in our models. Then, to account for having multiple recordings for individuals, we assigned unique identifiers to individuals and added it as a random model effect. Sex was also included as a fixed effect in the models to account for potential sex-based differences in CO_2_ exchange rates within each species. We checked for significant interactions between model effects, but none were found. Because the models without interaction terms had lower AICc values, no interactions were included in the final analysis.

The total flight distance and average velocity were also analysed separately for both species using linear models in JMP Pro version 16. Because we only used one clothianidin dose and collected data at a single time point, we analysed the data for this portion of the study by treatment group (control vs clothianidin-exposed) rather than by the calculated exposure level. All models included mass and treatment group, and for the monarch total distance model, the interaction between these terms, as it was found to be significant, and the AICc value of this model were the lowest. No other interactions were found to be significant for any other model and were therefore excluded.

## Results

**Figure 1:**
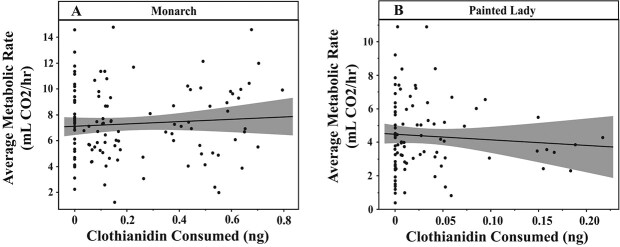
Average free-flight metabolic rates of monarch and painted lady butterflies exposed to clothianidin-treated and control solutions of artificial nectar. Average free-flight metabolic rate (ml CO_2_ h^−1^) across 5 minutes and the calculated amount of clothianidin consumed (ng) by (A) monarch and (B) painted lady butterflies exposed to artificial nectar treatments. Individuals fed the control treatments have a calculated clothianidin consumption of 0 ng. Shading indicates 95% confidence interval around the lines of best fit.

Clothianidin exposure decreased the average mass-corrected flight metabolic rates of painted lady butterflies (F_df = 1_ = 4.45, *P* = 0.039, n = 91), but not monarchs (F_df = 1_ = 0.21, *P* = 0.648, n = 120) (Fig. 1). Sex did not significantly affect average mass-corrected flight metabolic rates in either species (painted lady: F_df = 1_ = 0.01, *P* = 0.7664, n = 91| monarch: F_df = 1_ = 0.09, *P* = 0.944, n = 120).

**Figure 2:**
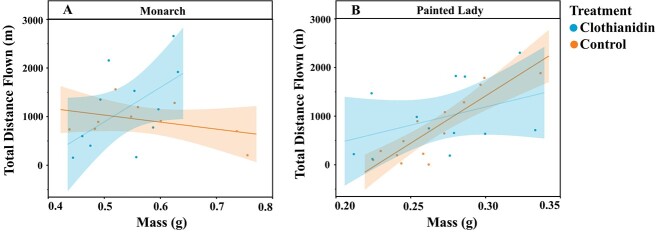
Total distance achieved on tethered flight mill by monarch and painted lady butterflies exposed to clothianidin-treated and control solutions of artificial nectar. Total tethered flight distance (m) and mass (g) of (A) monarch and (B) painted lady butterflies that were fed clothianidin-spiked and control artificial nectar solutions. Shading indicates 95% confidence interval around the lines of best fit.

For monarchs, there was a significant interaction between mass and clothianidin exposure that affected flight distance (F_df = 1_ = 4.87, *P* = 0.027, n = 21); indicating that for control butterflies, a larger mass was associated with a reduction in total flight distance, whereas for clothianidin-treated butterflies, a larger mass correlated with a longer flight distance (Fig. 2A). For painted ladies, mass was the only variable to significantly affect total flight distance (F_df = 1_ = 5.32, *P* = 0.021, n = 26), with larger butterflies flying longer distances than smaller individuals (Fig. 2B). Clothianidin exposure did not impact painted lady flight distance (F_df = 1_ = 0.01, *P* = 0.912, n = 26) (Fig. 2B).

**Figure 3:**
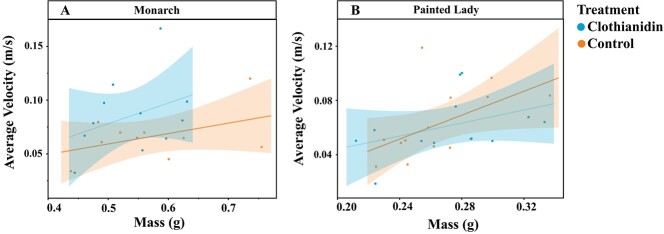
Average flight velocity achieved on a tethered flight mill by monarch and painted lady butterflies exposed to clothianidin-treated and control solutions of artificial nectar. Average tethered-flight velocity (m/s) and mass (g) of (A) monarch and (B) painted lady butterflies that were fed clothianidin-spiked and control artificial nectar solutions. Shading indicates 95% confidence interval around the lines of best fit.

For monarchs, clothianidin exposure (F_df = 1_ = 5.27, *P* = 0.022, n = 21), but not mass (F_df = 1_ = 2.58, *P* = 0.108, n = 21), impacted average flight velocity, with clothianidin-treated butterflies flying faster on average than control butterflies (Fig. 3A). For painted ladies, the average velocity results mirrored what we found for their total flight distances; mass was the only variable that significantly impacted average flight velocity (F_df = 1_ = 6.44, *P* = 0.011, n = 26), with larger butterflies flying faster on average than smaller butterflies (Fig. 3B). Again, there was no significant effect of clothianidin exposure (F_df = 1_ = 0.35, *P* = 0.556, n = 26) (Fig. 3B).

## Discussion

We sought to quantify the effects of adult neonicotinoid exposure on the flight performance of two species of migratory butterflies using respirometry and flight mill studies. In line with our prediction, we found that adult exposure to the neonicotinoid clothianidin lowered the mass-specific average flight metabolic rates of painted lady butterflies. Higher flight metabolic rates have been correlated with enhanced movement in other butterfly species (Niitepõld *et al.*, 2009); thus, our finding indicates that clothianidin exposure could harm the flight and dispersal potential of painted lady butterflies. However, contrary to what we expected, this result was not the same for both species. Adult clothianidin exposure did not significantly impact average flight metabolic rates in monarchs, suggesting that there may be differences in how sensitive these two species are to neonicotinoid compounds. It is possible that different feeding strategies (monarchs being milkweed specialists and painted ladies being broad generalists) may confer differences in their ability to excrete and/or detoxify neonicotinoid compounds. In general, it appears that monarchs exhibit relatively high tolerance to neonicotinoid exposure, particularly in comparison to honeybees (Krishnan *et al*., 2020), and it has been suggested that this may be the result of selection pressure for enhanced xenobiotic detoxification to allow for specialization on highly toxic host plants (Olaya-Arenas *et al*., 2020). However, it is unclear whether secondary metabolites, such as cardiac glycosides found in milkweed tissue, would result in cross-resistance to neonicotinoid insecticides.

Furthermore, other work from our lab has shown that when exposed to clothianidin-treated plants as larvae, monarch butterflies do exhibit significant decreases in flight metabolic rates (Cibotti *et al*., *in review*); however, in the present study, we did not observe any significant changes in flight metabolic rate in response to adult clothianidin exposure. This could indicate that monarch adults may be more resilient to neonicotinoid exposure than other life stages, a finding that would be consistent with toxicological assessments conducted on the species (Krishnan *et al*., 2021). However, a study by Wilcox *et al*. (2021) found no significant effects of larval clothianidin exposure on the rate of travel of radio-tagged adult monarchs, which could imply that monarch larvae are highly tolerant to neonicotinoid exposure as well.

In contrast with our results on free-flight energetics, tethered-flight velocity and distance were affected by clothianidin exposure in monarchs, but not painted ladies. Furthermore, the insecticide did not result in a reduction in these metrics for monarchs as we had expected. Clothianidin exposure only reduced the total flight distance of small individuals, whereas for large individuals it actually increased the total flight distance they were able to achieve. Additionally, clothianidin consumption increased the average flight velocity of monarchs of all sizes, rather than decreasing it as predicted.

While these results were unexpected, enhanced movement and activity in response to neonicotinoid exposure is not unprecedented. Acute exposure to sublethal doses of neonicotinoids is known to cause muscle hyperactivity in some insects (Camp and Buchwalter, 2016). After a high-dose exposure, muscle hyperactivity can manifest as severe muscle spasming and convulsions (Camp and Buchwalter, 2016), but low-dose exposures can, at least temporarily, stimulate learning and motor function (Lambin *et al*., 2001) and even increase insect movement speed under certain conditions (Crall *et al*., 2020). Therefore, the enhanced flight speeds and distances of clothianidin-treated monarchs that we observed in this study may be the result of this stimulation of motor activity.

It is possible that if we had either increased the time between the exposure event and the flight mill assay or exposed the butterflies for a longer period, we may have seen reductions in flight speed as predicted. However, another possibility is that the chosen doses were simply below the threshold required for flight inhibition. Some toxins and other stressors can elicit a biphasic dose–response relationship, in which low doses elicit stimulatory effects while high doses result in inhibition. This phenomenon is known as hormesis. While there are some who are critical of hormesis, particularly in regards to its potential application in regulatory and risk-assessment models (Crump, 2001; Kitchin and Drane, 2005; Mushak, 2013), there are also those who continue to document evidence of its occurrence across taxonomic groups and in response to a variety of stressors (Calabrese and Blain, 2005; Belz and Duke, 2014; Shibamoto and Nakamura, 2018; Agathokleous and Calabrese, 2021), including between insect pollinators and insecticides (Cutler *et al*., 2022). Since we only tested low-dose exposure levels in this study, we cannot conclude that our findings constitute evidence of a hormetic response to clothianidin exposure in monarch butterflies. However, we believe this could be an interesting framework to apply to future physiological assessments of neonicotinoid exposure in monarchs and other beneficial insect species.

When taken together, our results show that exposure to field-realistic levels of clothianidin in nectar can impact flight performance metrics in butterflies, but the results can vary both intra- and inter-specifically, depending on how flight performance is assessed. Our respirometry study revealed a negative impact of clothianidin exposure on painted lady average flight metabolic rate, whereas the flight mill study showed no effect of the insecticide on either the total flight distance or average flight velocity of painted ladies. Conversely, for monarch butterflies, the respirometry study showed no impact of clothianidin exposure on flight metabolic rates, whereas the flight mill results indicated a more stimulatory effect of the insecticide on flight. This highlights the fact that not all flight performance assays can be used interchangeably, as they each examine fundamentally different aspects of flight potential and come with their own sets of limitations. Being conducted under standardized laboratory conditions, neither flight assay accounts for important environmental factors (such as wind speed and direction, temperature and humidity ranges, weather events, etc.) that would likely have major impacts on flight performance in the wild. However, such factors aside, there are still several other limitations of each assay worth acknowledging.

While flow-through respirometry is a useful tool for examining respiratory exchange rates of organisms flying under standardized laboratory conditions, it can be difficult to interpret the results of these studies in a broad ecological context as they generally lack quantitative assessments of ‘effort’. Because of this, it is possible to interpret the results in a number of ways. For example, here we found that clothianidin exposure lowered mass-specific flight metabolic rates for painted ladies. This could indicate that clothianidin exposure actually increased the flight efficiency of the butterflies, as lower flight metabolic rates could imply a lower energetic cost of flight. However, it could also mean that the clothianidin-exposed butterflies were more lethargic and therefore exhibiting a lower flight effort. Unfortunately, since we did not have a simultaneous, quantitative assessment of flight effort, it is difficult to know which is true.

This limitation is partially why we initially sought to include flight mill evaluations in this study, to complement our flight respirometry data by providing additional insight on a different aspect of flight performance. However, flight mills also have several limitations in their ability to quantify flight capacity. Most of these limitations stem from the fact that flight mills evaluate effort on a tethered apparatus, where the organism is fixed to the arm of the mill and flying in a circular path rather than supporting its own weight while flying with a more natural directionality. This is an important distinction for several reasons. First, since the organism is fixed to the flight mill, it does not need to generate the lift required to remain airborne (Ribak *et al*., 2017; Naranjo, 2019). Second, forcing the insects to fly in a circular path can change their flapping kinematics, as they would naturally prefer to fly with a more linear directionality, and thus may flap asymmetrically and exert additional energy to try and veer off the circular path set by the mill (Ribak *et al*., 2017; Naranjo, 2019). Finally, while most modern flight mills, including those used in this study, are designed to minimize friction, they are not entirely frictionless. The insect therefore must generate more thrust to propel both itself and the flight mill forward than it would in free flight (Ribak *et al*., 2017). Combined, these differences between free- and tethered-flight assays are predicted to lead to significant differences in energetic requirements. Indeed, in *Manduca sexta* hawkmoths, oxygen consumption increases 2- to 5-fold between tethered and free flight (Heinrich, 1971).

In summary, our results indicate that low-level exposures to clothianidin-contaminated sugar solutions can impact flight performance metrics in both monarch and painted lady butterflies. Given prior work linking low flight metabolic rates with reduced dispersal capacity in butterflies (Niitepõld *et al*., 2009), our finding of a reduced average flight metabolic rate in painted lady butterflies in response to clothianidin consumption suggests that field-relevant levels of clothianidin exposure could negatively impact painted lady flight potential in the wild. However, we did not find strong evidence that exposure to low levels of clothianidin-contaminated sugar solution would hinder the flight performance of monarch butterflies. On the contrary, we observed a potential stimulatory effect of adult clothianidin exposure on monarch flight, though additional work would be required to assess how robust and long-lasting such effects may be. Although these findings alone may not serve as a crucial catalyst for conservation action, we believe that their contribution to the broader body of evidence regarding the impacts of these compounds on insect pollinators could ultimately help inform conservation efforts and research.

## Supplementary Material

Web_Material_coae002

## Data Availability

The data underlying this article will be shared on reasonable request to the corresponding author.
