# Supplementary material for: Divergent impacts of the neonicotinoid insecticide, clothianidin, on flight performance metrics in two species of migratory butterflies
Source: Conserv Physiol. 2024 Feb 2;12(1):coae002. doi: 10.1093/conphys/coae002 (PMC10836301; doi:10.1093/conphys/coae002)
Supplement: Web_Material_coae002 [file web_material_coae002.pdf]

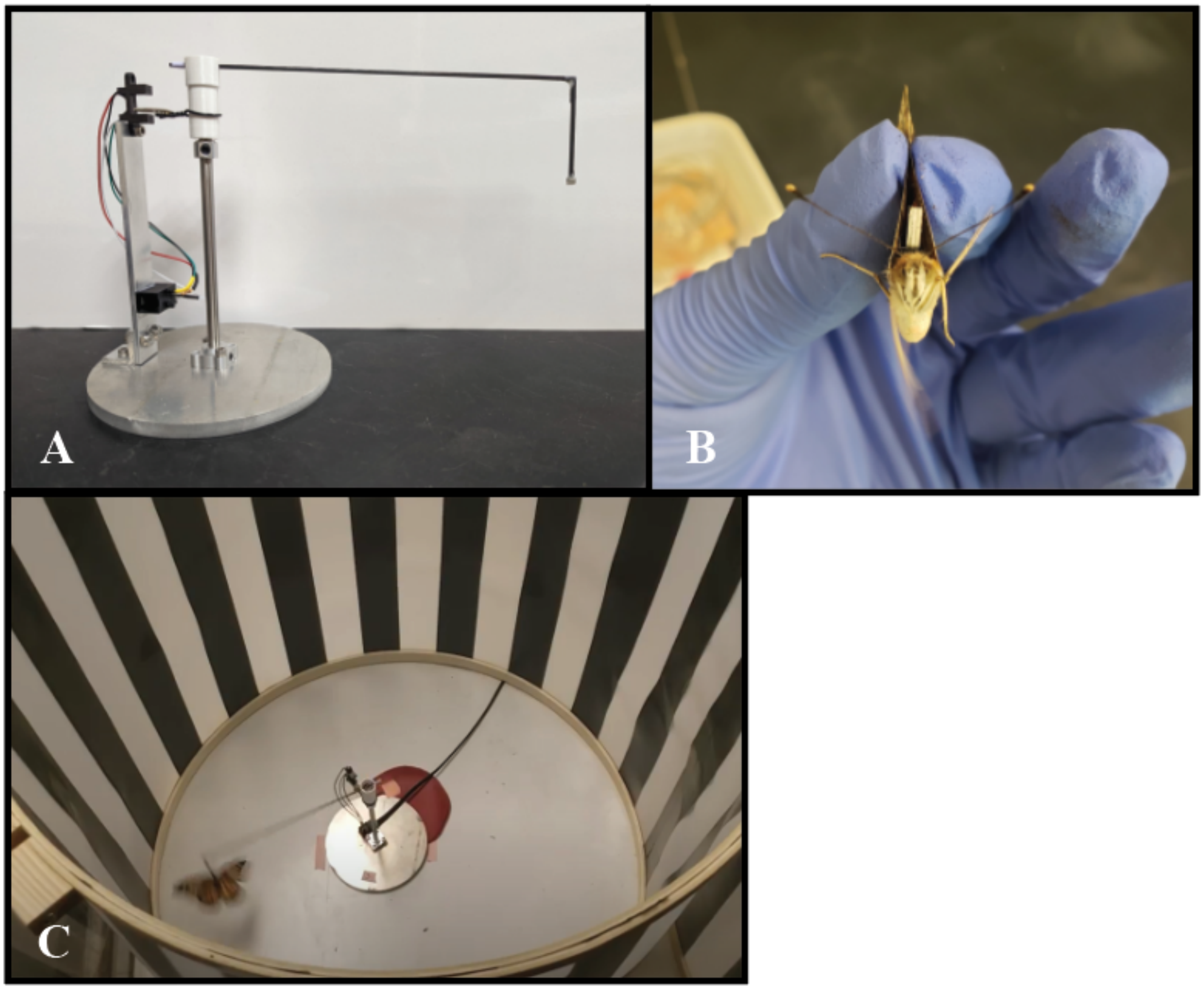

Supplementary Figure 1. Photos of the custom-designed flight mills and insect attachments used in this study. (A) The flight mill stand, detector, and arm. Missing from this photo (but shown in panel C) is the cord which connected the 4P4C modular jack near the base of the flight mill to a Mega 2560 board, allowing for data to be sent from the flight mill detector to a PC where it could be recorded and stored. (B) A *Vanessa cardui* (painted lady) butterfly with an L-shaped metal tab adhered to its thorax. This metal tab attached to the square magnet on the arm of the flight mill to keep the butterfly affixed to the mill during the flight mill assay. (C) A *Danaus plexippus* (monarch) butterfly attached to the flight mill and flying in circuit. The mill is

surrounded by a tube-shaped sheet of plastic which reduced draft and other external stimuli. The black and white striping provided the butterfly with a sense of optical flow during flight.

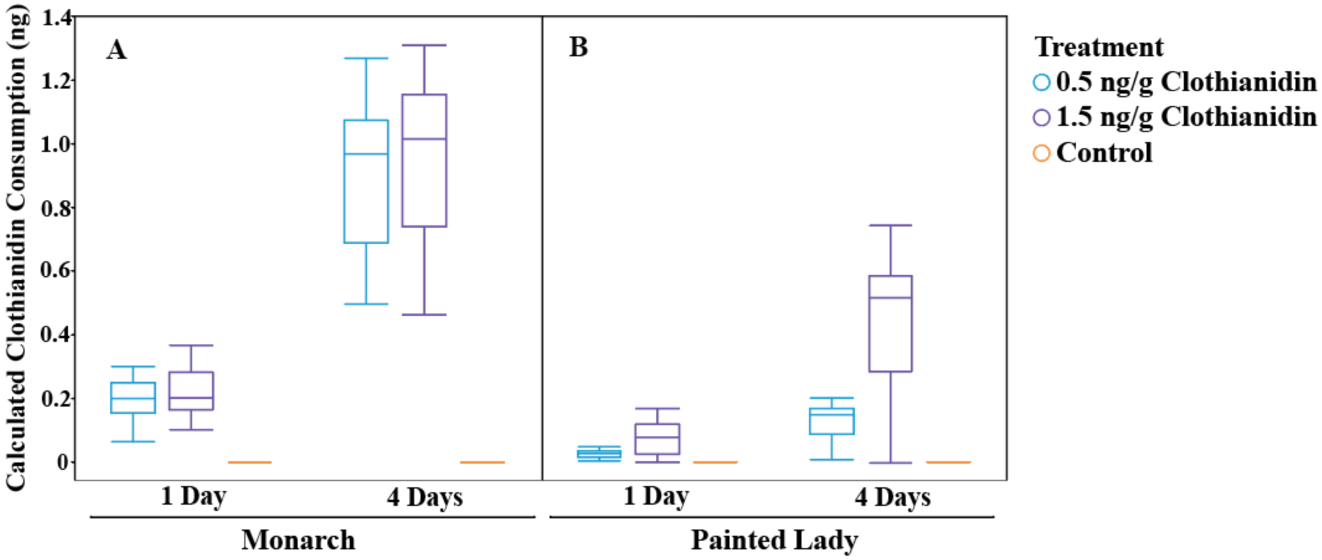

Supplementary Figure 2. Calculated amount of clothianidin consumed by monarch and painted lady butterflies exposed to clothianidin-treated and control solutions of artificial nectar. Boxplots displaying the calculated amount of clothianidin consumed (ng) by (A) monarch and (B) painted lady butterflies after feeding on artificial nectar solutions containing 0.5 ng/g clothianidin (blue), 1.5 ng/g clothianidin (purple), or 0 ng/g clothianidin (orange) for either 1 day or 4 consecutive days.
